# The contribution of stimulus frequency and recency to set-size effects

**DOI:** 10.3758/s13423-017-1342-4

**Published:** 2017-12-05

**Authors:** Félice van ‘t Wout

**Affiliations:** 1 University of Exeter, Exeter, UK; 2 Present Address: School of Experimental Psychology, University of Bristol, Bristol, BS8 1TH England

**Keywords:** Hick’s law, Memory retrieval, Set-size effect

## Abstract

Hick’s law describes the increase in choice reaction time (RT) with the number of stimulus-response (S-R) mappings. However, in choice RT experiments, set-size is typically confounded with stimulus recency and frequency: With a smaller set-size, each stimulus occurs on average more frequently and more recently than with a larger set-size. To determine to what extent stimulus recency and frequency contribute to the set-size effect, stimulus set-size was manipulated independently of stimulus recency and frequency, by keeping recency and frequency constant for a subset of the stimuli. Although this substantially reduced the set-size effect (by approximately two-thirds for these stimuli), it did not eliminate it. Thus, the time required to retrieve an S-R mapping from memory is (at least in part) determined by the number of alternatives. In contrast, a recent task switching study (Van ‘t Wout et al. in *Journal of Experimental Psychology: Learning, Memory & Cognition., 41*, 363–376, 2015) using the same manipulation found that the time required to retrieve a task-set from memory is not influenced by the number of alternatives per se. Hence, this experiment further supports a distinction between two levels of representation in task-set control: The level of task-sets, and the level of S-R mappings.

According to Hick’s law, reaction time (RT) increases as a function of the number of stimulus-response (S-R) mappings (Hick, 1952; Hyman, 1953), approximately linearly with the logarithm of the number of alternatives. This may be seen as an example of the more general finding that the difficulty of memory retrieval increases with the number of competing items in memory. Other examples of such a “set-size effect” include the “fan effect” (Anderson, 1974) and the decrease in free recall performance with list length (Murdock, 1962). However, one feature of such paradigms is that increasing set-size usually results in each item being tested less frequently and hence less recently. As recency and frequency are likely to influence retrieval time (Anderson, 1976), it is possible that set-size effects reflect (at least in part) effects of stimulus recency and frequency. The purpose of this experiment was to examine the contribution of stimulus recency and frequency to the set-size effect found in choice RT experiments.

This experiment was motivated in part by a recent task-switching study, which investigated whether the effect of the number of competing items on retrieval time also applies to the number of tasks among which a participant must switch (Van ‘t Wout, Lavric & Monsell, 2015). In this study participants were required to switch among three or five tasks. Overall, the five-task condition yielded longer RTs and switch costs, especially with no time to prepare for the upcoming trial. For two of the tasks, the frequency (and hence, recency) with which they were encountered was matched between the five-task and the three-task condition. For those tasks, the number of tasks among which participants had to switch in a block of trials did not influence performance. Hence the effect of number of tasks on the other tasks was not in fact attributable to a greater difficulty in task-set activation when more tasks were in play.

Van ‘t Wout et al.’s (2015) finding that task-set retrieval is not influenced by the number of alternatives is in contrast with the common observation that memory retrieval increases with the number of alternatives. There are two possible explanations: 1) the process of retrieving a task-set in a task-cueing environment is somehow “special” and differs from other kinds of memory retrieval in this respect; or 2) set-size effects observed for other kinds of memory retrieval actually might also result from the confound with the recency and/or frequency of retrieval, not from set-size per se. The experiment reported here tested the latter explanation for the set-size effect found in choice RT experiments (Hick, 1952; Hyman, 1953).

In a typical set-size experiment, participants are required to execute a varying number of arbitrary S-R associations in different blocks. Hick’s law describes the increase in RT with the number of S-R mappings. According to Schneider and Anderson’s (2011) memory-based model of Hick’s law, one source of this set-size effect is associative interference during retrieval. The idea that Hick’s law can be explained in terms of basic memory effects is supported by studies that have shown that set-size effects are not found when the need to retrieve arbitrary S-R associations is eliminated. Examples of such studies include experiments involving saccades to a target location (Kveraga, Boucher & Hughes, 2002), aimed arm movements (Wright, Marino, Belovsky & Chubb, 2007), and the naming of very familiar stimuli, such as letters (Morin, Konick, Troxell & McPherson, 1965). In contrast, others have found evidence of set-size effects even when memory load was constant across set-size conditions (anti-saccades in Kveraga, Boucher & Hughes, 2002; Brown, Steyvers & Wagenmakers, 2009), arguing against a purely memory-based model of Hick’s law.

Another factor known to influence set-size effects is the frequency of immediate response repetitions on successive trials. As Kornblum (1968) noted, there is an inverse correlation between the number of S-R mappings and the probability of an immediate S-R repeat. Because RTs are typically faster for an S-R repeat than for an S-R switch (Bertelson, 1961), decreasing the number of S-R mappings inflates the proportion of fast(er) responses in the RT distribution. Kornblum (1968) tested this prediction by varying the number of S-R repetitions independent of set-size and found that RTs increased as a function of set-size only when the probability of an S-R repetition was high.

However, less appears to be known about the effect of stimulus recency beyond immediate response repetitions on Hick’s law. Although Hyman (1953) did find a positive correlation between RT and stimulus frequency (Experiment 2) and recency (Experiment 3) in his original paper, the interaction between these variables was not further investigated. In other words, Hyman’s findings did not tell us whether, if recency and frequency were matched, the set-size effect would disappear. The experiment reported was aimed at investigating whether this was indeed the case. This experiment additionally investigated the effect of practice on set-size effects, as previous research into the role of practice has been inconclusive: Whereas some have argued that set-size effects might disappear with vast amounts of practice (Teichner & Krebs, 1974), others have found that a substantial set-size effect remains even after extensive practice (for example, both Hale, 1968, and Hyman, 1953, reported sizeable set-size effects after 5000 and 15000 trials, respectively).

In summary, it appears at least possible that the set-size effects found in choice RT experiments are (in part) the result of the frequency and recency with which the specific S-R retrieval has been exercised. To assess this possibility, this experiment manipulated S-R frequency, recency, and set-size in a choice RT experiment much as van’t Wout et al. (2015) did for tasks. To achieve this, participants were required to classify a set of 4 stimuli with 4 responses in some blocks, and a set of 6 stimuli with 6 responses in others. Two different sets of stimuli were used: a set of colours, and a set of shapes, so that half of the participants classified 4 colours, and 6 shapes, and vice versa for the other half of participants. For 2 of those stimuli (which we will refer to as “probe” stimuli), recency and frequency were matched between the 4 S-R and the 6 S-R conditions. If the set-size effect really results from a confound between set-size and recency or frequency, then a set-size effect should be found for the nonprobe stimuli but not for the probe stimuli for which recency and frequency did not differ between the 4 and 6 S-R conditions.

## Method

### Participants

Twenty-four participants (aged between 18 and 45 years, M = 21.6, 22 females and 2 males) took part in this experiment. All provided informed consent prior to participating, and the experiment was approved by the University of Exeter School of Psychology Ethics Committee. Participants were paid between £5.40 and £7.00, depending on the speed and accuracy of their performance.

### Design and procedure

To manipulate the number of S-R mappings within subjects between the two halves of the experiment, whilst avoiding any impact of previous exposure to the same stimuli on performance in the second half, 2 sets of 6 stimuli were used: a set of 6 colours (green, red, light blue, purple, yellow, and dark blue), and a set of 6 shapes (circle, cross, drop, square, star, and triangle). Responses were made using (4 or all) the X, C, V, B, N, and M keys on a computer key board, pressed with the ring, middle, and index fingers of the left and right hands. Half the participants completed the shape task with 4 S-R mappings and the colour task with 6 S-R mappings, and vice versa for the other half of participants. In this way, the experiment was split into two parts (a 6 S-R and a 4 S-R part), and the order of parts (and tasks) was balanced between participants.

**Fig. 1:**
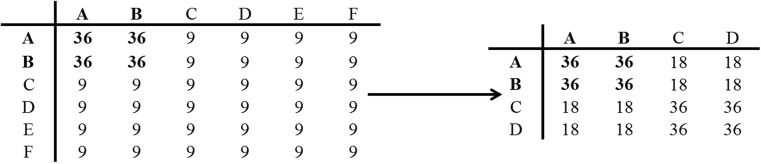
Trial matrix displaying the frequency of all transition types in the 6 S-R condition (left) and the 4 S-R condition (right), with the probe transitions highlighted in bold

Each part was split up into 9 blocks of 48 trials each, plus 1 warm-up trial. For 2 of the S-R mappings (the “probe” mappings), recency and frequency were matched between the 4 S-R and 6 S-R conditions. This was achieved by yoking one participant (P_1_) with another participant (P_2_). First, a 6 S-R sequence (for participant P_1_) was created, in which the probe transitions (AA, BB, AB, and BA) occurred 4 times as often as all the other (nonprobe) transitions (Fig. 1). In order to create the 4 S-R sequence (for participant P_2_), all Es and Fs were replaced with Cs and Ds, respectively. In this manner, in the 4 S-R sequence, all 4 probe transitions (and the CC, DD, CD, and DC nonprobe transitions) occurred twice as often as the other nonprobe transitions. So, stimulus recency was matched for probe stimuli between (yoked) subjects, and frequency also was matched within subjects.

The order of number of S-R mappings (in the two halves of the session) and the order of stimulus set used for each half was manipulated between subjects. Furthermore, in the 4 S-R condition each participant was assigned one of three response sets, consisting of two responses of the left and right hands: 1) ring and middle finger; 2) middle and index finger; and 3) index and ring finger. This was done so that between subjects, each response was made equally often in the 4 and 6 S-R condition.

Prior to the start of each of the 4 S-R and 6 S-R parts, participants completed 1 practice block of 48 trials (plus 1 warm-up trial). In both the practice and the experimental sessions, the trial sequence was as follows: a 500-ms blank interval between trials, followed by a 500-ms fixation dot, after which the stimulus appeared. The stimulus remained on the screen until a response was made. On incorrect trials, an error message remained on the screen for 1,000 ms until the trial sequence resumed. The 4 S-R and 6 S-R parts both consisted of 9 blocks of 48 trials (plus 1 warm-up trial) each, and the parts were separated by a 5-minute break. At the end of each block, participants were presented with a score that was based on the speed and accuracy of their responses. When participants improved on this score, a bonus point (£0.10 each) was awarded. In total, the session lasted approximately 50 minutes.

## Results

Very long (>2,000 ms) and short (<200 ms) reaction times (RTs), trials following an error and warm-up trials were excluded from the data set (0.5% of the correct responses). Furthermore, immediate response repetitions were excluded from analysis (Kornblum, 1968) except for the analysis of the effect of recency. As a result, the probe task trials analysed were either AB or BA transitions (16.67% of all trials). Because both of these trial types involved a hand switch, hand repeats were excluded from the analysis of the nonprobe trials.

### Set-size effects

**Fig. 2:**
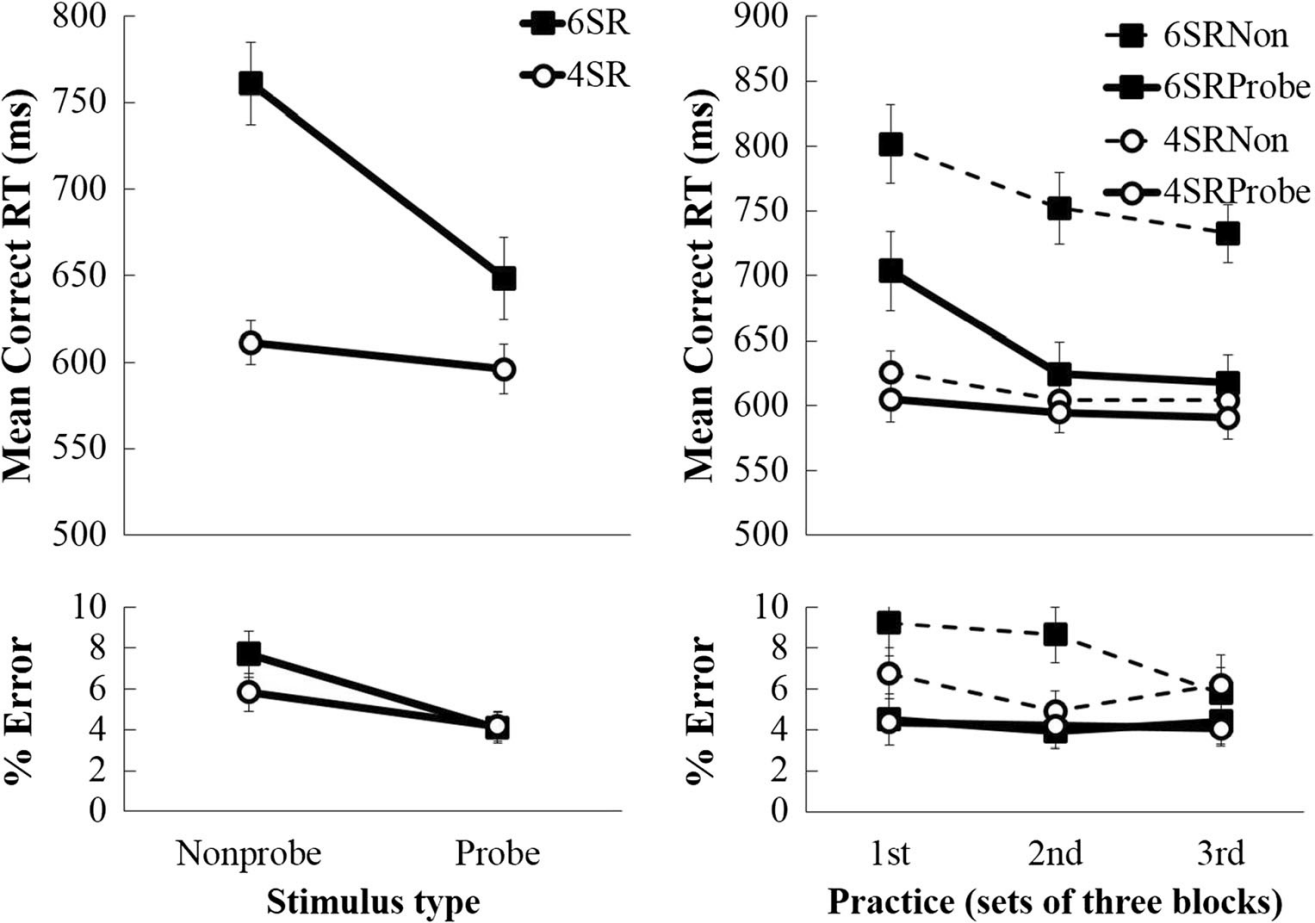
Mean correct RT (top) and % error (bottom) data, for 6 S-R and 4 S-R trials, plotted as a function of probe/nonprobe stimuli (left), and as a function of practice (right)

Set-size effects for probe and nonprobe stimuli are shown in Fig. 2 (left). A 2 (4 S-R or 6 S-R) x 2 (probe or nonprobe) repeated measures ANOVA was run on the mean correct RT data to compare set-size effects for probe trials (following probe trials) and nonprobe trials (following nonprobe trials). Overall, this analysis revealed a significant set-size effect of 101 ± 17 ms,^1^ F(1,23) = 36.94, *p* < 0.001, *η*_*p*_^2^ = 0.616. A significant two-way interaction demonstrated that the set-size effect was much larger for the nonprobe stimuli (150 ± 20 ms) than for the probe stimuli (52 ± 22 ms), F(1,23) = 13.48, *p* = 0.001, *η*_*p*_^2^ = 0.370. RTs also were shorter for probe stimuli (622 ± 16 ms) than for nonprobe stimuli (686 ± 16 ms), F(1,23) = 19.65, *p* < 0.001, *η*_*p*_^2^ = 0.461. Additional ANOVAs revealed that the set-size effect was significant both for probe stimuli, F(1,23) = 5.56, *p* = 0.027, *η*_*p*_^2^ = 0.195, and for nonprobe stimuli, F(1,23) = 53.74, *p* < 0.001, *η*_*p*_^2^ = 0.700. Hence, although matching for recency and frequency reduced the set-size effect considerably (by 65%), a significant set-size effect still remained for probe stimuli, suggesting that the effect cannot be entirely attributed to stimulus recency or frequency. 2

Participants also made slightly more errors in the 6 S-R (5.9 ± 0.7%) compared with the 4 S-R condition (5.0 ± 0.7%), although this difference was not significant, F(1,23) = 1.33, *p* = 0.261, *η*_*p*_^2^ = 0.055. They made significantly fewer errors on probe (4.1 ± 0.6%) than on nonprobe (6.8 ± 0.8%) trials, F(1,23) = 12.32, *p* = 0.002, *η*_*p*_^2^ = 0.349. Although, as for RTs, the set-size effect was larger for nonprobe (1.9 ± 1.2%) than for probe stimuli (−0.1 ± 1.0%), the two-way interaction was not significant, F(1,23) = 1.64, *p* = 0.213, *η*_*p*_^2^ = 0.067.

### Modulation of set-size effect by practice

To investigate potential effects of practice on the set-size effect, the data were split up into 3 parts of 144 trials each (first 3 blocks vs. second 3 blocks vs. third 3 blocks. Note that the sequential constraints described in the *Method* section were applied per block trio, so that each part contained equal numbers of all stimuli, transition types, etc.). A 3 (practice) x 2 (4 S-R or 6 S-R) x 2 (probe or nonprobe) repeated measures ANOVA was run on the mean RT and % error data. Only significant interactions with the linear component of the effect of practice are reported.

Overall, the effect of set-size was significantly reduced with practice (from 137 ± 24 ms in the first part to 78 ± 16 ms in the third part, a reduction of 59 ms or 43%), F(1,23) = 8.06, *p* = 0.009, *η*_*p*_^2^ = 0.259. This reduction in set-size effect with practice was numerically greater for the probe stimuli (from 99 ± 27 ms to 27 ± 21 ms, a reduction of 72 ms or 73%) than for the nonprobe stimuli (from 176 ± 30 ms to 128 ± 22 ms, a reduction of 47 ms or 27%), although the three-way interaction between probe, number, and practice was not significant, F(1,23) = 0.87, *p* = 0.362, *η*_*p*_^2^ = 0.036.The reduction in set-size effect with practice is interesting, because it might suggest that, perhaps with more practice, the set-size effect could disappear altogether. Indeed, for probe trials, when the first part was excluded from the analysis, the set-size effect was no longer statistically significant, F(1,23) = 1.68, *p* = 0.208, *η*_*p*_^2^ = 0.068. However, it was nontrivial in magnitude (28 ms) and appears asymptotic.

### Recency analysis

**Fig. 3:**
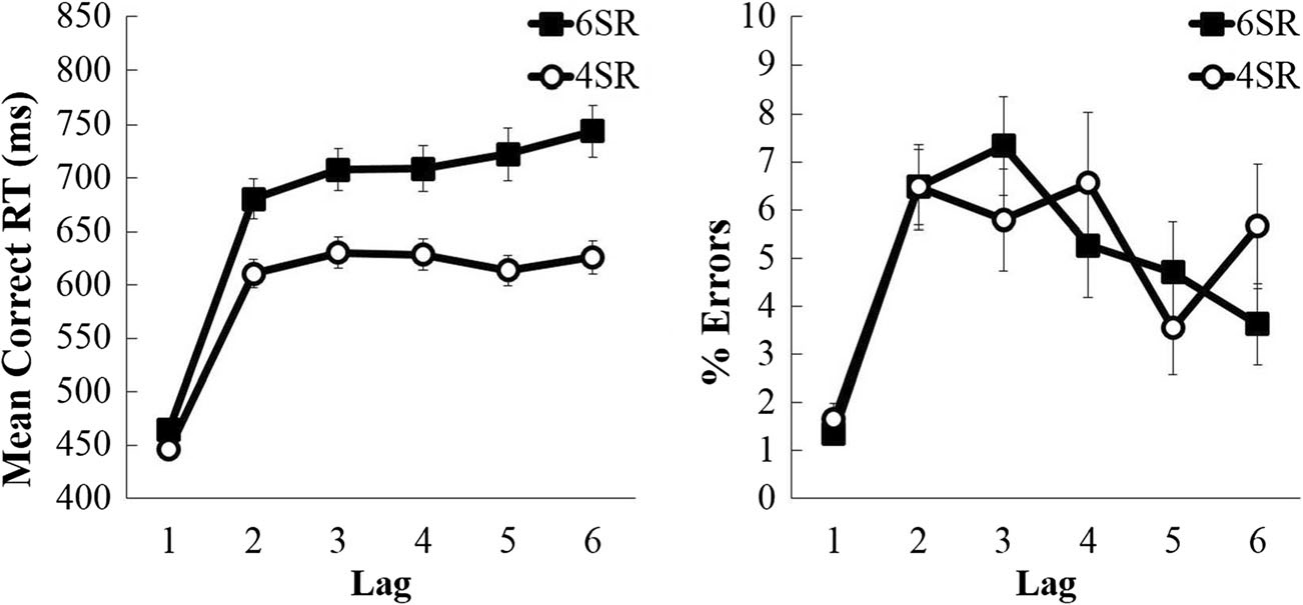
Mean correct RT (left) and % error (right) data for 6 S-R and 4 S-R trials, plotted as a function of lag

RT and errors also were analysed as a function of lag: the number of trials since the previous appearance of the present stimulus. This analysis was restricted to trials with a lag up to 6 (with lag 1 being an immediate S-R repeat). The analysis included all probe and all nonprobe trials (there was not enough data to restrict this analysis to probe-probe and nonprobe-nonprobe sequences). The first thing to notice from Fig. 3 is the massive (190 ± 12 ms, F(1,23) = 267.64, *p* < 0.001, *η*_*p*_^2^ = 0.921) increase in RT from lag 1 to lag 2. That is, most of the effect of lag was due to immediate repetitions. This increase also was significantly larger in the 6 S-R condition (216 ± 17 ms) than in the 4 S-R condition (164 ± 10 ms), F(1,23) = 9.77, *p* = 0.005, *η*_*p*_^2^ = 0.298.

Of interest is whether RTs continued to increase beyond lag 2. In a further analysis, lag 1 trials were excluded from the analysis. Only effects of and interactions with the linear component of recency will be reported below. There was a small (slope 8 ms) but significant recency effect beyond lag 2, F(1,23) = 9.47, *p* = 0.005, *η*_*p*_^2^ = 0.292. This recency effect is only present in the 6 S-R condition (slope 14 ± 4 ms, F(1,23) = 10.37, *p* = 0.004, *η*_*p*_^2^ = 0.311), and not in the 4 S-R condition, (slope 1 ± 2 ms, F = 0.39, *p* = 0.539, *η*_*p*_^2^ = 0.017.); the difference between conditions in the magnitude of the recency effects beyond lag 2 was significant, F(1,23) = 6.81, *p* = 0.016, *η*_*p*_^2^ = 0.229.

However, this pattern is not replicated in the error data. Although the error rates increased substantially (by 5.0 ± 0.6%) from lag 1 to lag 2, F(1,23) = 65.87, *p* < 0.001, *η*_*p*_^2^ = 0.741, this increase was not significantly larger in the 6 S-R (5.1 ± 0.8%) than in the 4 S-R (4.8 ± 0.8%) condition, F(1,23)=.54, p=.819, *η*_*p*_^2^ =.002. Furthermore, beyond lag 2, the error lag effect opposed the RT lag effect: participants made fewer errors (slope −0.6 ± 0.2%) with an increase in lag, F(1,23) = 13.63, *p* = 0.001, *η*_*p*_^2^ = 0.372.

Most importantly, the difference in slope between the 6 S-R and 4 S-R condition demonstrates that unequal proportions of more recent trials alone cannot explain the occurrence of set-size effects; had this been the case, there should have been no difference whatsoever between the 4 S-R and 6 S-R conditions once the data are plotted as a function of recency.

### Frequency analysis

The large effect of set-size for the nonprobe items, coupled with the absence of a marked recency effects beyond lag 1 suggests frequency is important. The effect of frequency can be assessed independently of the number of S-R mappings, because in the 6 S-R condition, probe stimuli were responded to more frequently (108 times each per session) than nonprobe stimuli (54 times each per session; Fig. 1). These more frequent probe stimuli were responded to 96 ± 27 ms faster than the less frequent nonprobe stimuli, F(1,23) = 12.88, p = 0.002, *η*_*p*_^2^ = 0.359. The same trend was apparent in the errors (7.9 ± 0.9% less frequent stimuli; 4.0 ± 0.7% more frequent stimuli), F(1,23) = 16.58, *p* < 0.001, *η*_*p*_^2^ = 0.419. Because this analysis of the frequency effect was overall confounded with stimulus recency, the same analysis was run with the data binned by lag (restricted to lag positions 2 to 6, hence not including immediate stimulus repetitions) and then averaging over lag. This estimate of the frequency effect could not be affected by an inflated proportion of more recent stimuli for the more frequent stimuli. The result was similar: RTs remained faster for the more frequent (681 ± 23 ms) than the less frequent (744 ± 23 ms) stimuli, F(1,23) = 8.40, p = 0.008, *η*_*p*_^2^ = 0.286. This frequency analysis confirms that frequency influences RT independently of set-size.

## Discussion

This experiment was designed to determine the contribution of stimulus recency and frequency to the set-size effect found in choice RT tasks. Participants were required to identify six colours and four shapes (or vice versa), in two separate parts of the experiment. For two of the stimuli in each part (the “probe” stimuli), frequency and recency of usage were matched between parts. It was predicted that if the set-size effect merely describes an effect of recency and/or frequency, not an effect of set-size per se, then no set-size effect should be observed for the probe stimuli. The results did not confirm this prediction. Although the set-size effect was approximately 3 times larger for nonprobe stimuli compared with probe stimuli, a significant overall set-size effect of 52 ms remained for probe stimuli. This finding suggests that although stimulus frequency and/or recency are a source of set-size effects in “uncontrolled” data sets, they are not the only source. Further analyses showed that immediate stimulus repetitions substantially contribute to set-size effects, as does stimulus frequency. Stimulus recency beyond immediate repetitions did not appear to have much of an effect. Altogether, the analyses clearly demonstrated that even when recency and frequency of usage are matched, retrieving an S-R mapping from among alternatives is influenced by the number of competitors.

These results are consistent with Schneider and Anderson’s (2011) memory-based model of Hick’s law, in which a chunk’s base-level activation reflects frequency and recency of usage. Although other models (such as Brown et al.’s (2009) evidence accumulation model, or Usher, Olami, & McClelland’s (2002) model, which views the set-size effect as a speed accuracy trade-off) also are able to produce set-size effects, it is not obvious how these models could account for the effects of recency and frequency observed here.

As it has previously been demonstrated that practice modulates set-size effects (Hale, 1968; also see Longstreth, El-Zahar & Alcorn, 1985), the data also were analysed as a function of practice. Consistent with previous findings, this analysis revealed that overall, the set-size effect decreased as a function of practice. For the nonprobe stimuli, the set-size effect decreased by 27% in the last third of the half-session compared with the first third. For the probe stimuli, however, this reduction was much larger (73%). Indeed, when the first third was removed from the analysis, the set-size effect for probe trials was no longer significant. However, at 28 ms, the set-size effect, although nonsignificant, had still not disappeared entirely and appeared asymptotic.

The set-size effect obtained for probe trials in this experiment can be contrasted with the results of Van ‘t Wout et al. (2015), in which the effect of the number of alternative task-sets (three or five) on task switching performance disappeared when the tasks were matched for recency and frequency. The results of that study demonstrate that retrieving a task-set from memory is not influenced by the number of alternative task-sets. In contrast, the data reported here show that retrieving an S-R mapping from memory *is* influenced by the number of alternatives. This apparent discrepancy demonstrates that the process of selecting one S-R mapping amongst alternatives (this experiment), and the process of retrieving one task-set amongst others (Van ‘t Wout et al., 2015) are not subject to the same constraints or capacity limits. Together, the results of these studies are consistent with an account of procedural memory which distinguishes between a component of memory holding all potentially relevant task-sets, with the task-sets in play in the current block of trials in an active state, with no limit on the total pool of activation (or none that affects retrieval time), and a capacity limited component, holding only the currently operative task-set, as proposed by Oberauer (2009). Such a theory makes two predictions: 1) The time consumed by a task change is not influenced by the number of other task-sets, because these are represented in the (capacity unlimited) part of long term memory (Van ‘t Wout et al., 2015); and 2) The time required to select the appropriate S-R mapping should increase as a function of the number of competitors (within the same task-set), because the currently operative task-set is held in a capacity limited buffer. The results reported confirm this second prediction, and hence they provide an additional reason for distinguishing between the level at which task-sets (packages of S-R rules) are represented and the level at which S-R rules are represented.

## Footnotes

1 Plus or minus symbol (±) indicates the SEM difference.

2 This analysis was restricted to probe-probe and nonprobe-nonprobe sequences in case performance also was influenced by the recency/frequency of the immediately preceding trial. Without restrictions on the preceding trial type, the set-size effect was still much larger for the nonprobe stimuli (142 ± 20 ms, F(1,23) = 48.31, *p* < 0.001, *η*_*p*_^2^ = 0.677) than for the probe stimuli (67 ± 20 ms, F(1,23) = 10.91, *p* = 0.003, *η*_*p*_^2^ = 0.322), F(1,23) = 8.19, *p* = 0.009, *η*_*p*_^2^ = 0.263, although this difference is somewhat smaller (the effect for nonprobe exceeds that for probe stimuli by a factor of 2 rather than 3). This suggests frequency/recency of the preceding trial may matter; hence the main analysis was restricted to probe-probe and nonprobe-nonprobe sequences.
